# Bio inspired assessment of titanium-organic framework and exosome-constructed p-Synephrine carriage: pursuing the PI3K/mTOR pathway in a simulated periodontitis

**DOI:** 10.1038/s41598-026-54070-6

**Published:** 2026-05-25

**Authors:** Mohamed S. Kishta, Abdelrahman H. Elshaar, Rawan N. Makled, Ghada A. Abdel-Aleem, Mirhan N. Makled, Reda M. Abdelhameed

**Affiliations:** 1 Researcher of medical biotechnology, Hormones Department, Medical Research and Clinical Studies Institute, National Research Centre, 33 El Buhouth St., El Dokki, P.O.B: 12622, Cairo, Egypt; 2 Faculty of Oral and Dental Medicine, Future University, Cairo, Egypt; 3 Mansoura Manchester Dental programme, Faculty of Dentistry, Mansoura University, Mansoura, Egypt; 4 Medical Biochemistry Department, Faculty of Medicine, Tanta University, El Gharbia, Egypt; 5 Pharmacology and Toxicology Department, Faculty of Pharmacy, Mansoura University, Mansoura, Egypt; 6 Applied Organic Chemistry Department, Chemical Industries Research Institute, National Research Centre, 33 EL Buhouth St.,Dokki, Giza, 12311 Egypt

**Keywords:** Periodontitis, MIL-125, p-Synephrine, Exosomes, Dental pulp stem cells (DPSCs), Cell biology, Diseases, Drug discovery, Medical research

## Abstract

Periodontitis, a chronic inflammatory disease, is driven by bacterial infection and oxidative stress, leading to tissue destruction and potential tooth loss. This study investigates the anti-inflammatory and antioxidant potential of p-Synephrine and enhanced delivery through NH_2−_MIL-125 and exosomes derived from dental pulp stem cells (DPSCs). Primary Normal Human Gingival Keratinocytes (PCS) and Gingival Fibroblasts (HGF) were divided into eight groups, including controls, induction with LPS, and treatments with NH_2−_MIL-125, exosomes, free p-Synephrine, p-Synephrine-loaded NH_2−_MIL-125 (P-SYN-NH_2−_MIL-125), p-Synephrine-loaded exosomes (P-SYN-Exo), and dexamethasone as a reference drug. Pro-inflammatory cytokines (IL-4, IL-6, TNF-α) and pathway markers (PI3K and mTOR) were quantified using ELISA kits, while antioxidant enzyme activities (GPx, SOD, and TAC) were assessed using colorimetric assays. Results showed that p-Synephrine loaded into NH_2−_MIL-125 reduced inflammation markers and enhanced antioxidant defenses by increasingof GPx, SOD, and TAC concentrations. Among all treatments, p-Synephrine-loaded exosomes (P-SYN-Exo) demonstrated the most significant results, showing the highest increase in antioxidant markers GPx, SOD, and TAC, alongside a pronounced reduction in pro-inflammatory cytokines IL-4, IL-6, and TNF-α. Furthermore, p-Syn-Exo exhibited the most marked decrease in signaling pathway markers PI3K and mTOR. NH_2−_MIL-125 and exosomes amplified these effects through controlled release and improved bioavailability, demonstrating superior reductions in TNF-α, IL-4, and IL-6 and increased antioxidative stress markers. These findings highlight p-Synephrine, particularly when delivered via NH_2−_MIL-125 and exosomes, as a promising adjunctive treatment for periodontal inflammation and oxidative stress.

## Introduction

Inflammation is the body’s natural response to injury or infection, involving the release of immune chemicals to heal damaged tissue, but chronic inflammation occurs when this response persists inappropriately, leading to damage of healthy cells and tissues, and increasing the risk of various diseases^1^. Periodontal tissue is characterized by a chronic inflammation of the gingival tissues and results from the accumulation of microbial plaque on the tooth surface due to inadequate brushing Some of the genera of pathogenic bacteria include, but are not limited to, *phyla Bacteroidetes*,* Firmicutes*,* Proteobacteria*,* Spirochaetes and Synergistetes*^2^ most of them when invading cause inflammation in the gingiva which consequently causes oxidative stress in the cells^3^. Periodontal disease led to an estimated $154.06 billion loss in the United States and €158.64 billion in Europe in 2018^4^, reflecting both direct treatment costs and indirect burdens like lost productivity, reduced quality of life, and added healthcare expenses, underscoring the need for preventative care and early intervention.

Effective brushing practices are therefore essential to remove food debris and inhibit further plaque development. Periodontitis is classified as localized when bleeding upon periodontal probing affects approximately 30% or less of the gingival tissue, and as generalized if more than 30% is affected^5^. Periodontitis and other oral infections can cause inflammation, tissue destruction, and tooth loss, with treatment options ranging from nonsurgical to surgical methods, though no single approach is superior; effective management involves advanced techniques to reduce disease progression, with oral hygiene playing a key role in minimizing plaque buildup and gingivitis^6^. Supportive therapy, including professional mechanical plaque removal (PMPR), can help reduce the frequency and annual rate of tooth loss and clinical attachment loss in periodontitis patients. However, the long-term effects of this intervention on periodontal health indicators remain to be fully understood^7^. Commercially available local antimicrobials can be beneficial in certain clinical situations, such as treating localized deep pockets or during supportive periodontal therapy^8^. However, their effectiveness is limited by an uncertain cost–benefit ratio. Systemic antimicrobials are not suitable as standalone treatments for periodontitis; however, combinations such as amoxicillin with metronidazole, ciprofloxacin with metronidazole, metronidazole alone, or azithromycin may enhance the clinical outcomes of nonsurgical periodontal therapy, particularly in cases of aggressive, severe, or recurrent periodontitis^9^. Despite these benefits, systemic antimicrobials carry the risk of adverse effects and contribute to bacterial resistance, necessitating cautious use.

Under normal conditions, antioxidants neutralize reactive oxygen species (ROS) to prevent tissue damage, but in inflammatory diseases like periodontitis, ROS levels rise sharply due to immune cell activity, particularly through neutrophil “respiratory bursts” during phagocytosis^10^. Periodontitis is linked to heightened activity in peripheral blood neutrophils, which are considered the primary source of reactive oxygen species (ROS) in this condition also recent studies support this and propose that this neutrophil hyperactivity may represent a host immune response aimed at combating the inflammatory process associated with periodontitis^11^. The balance between ROS production and antioxidant defenses is critical for health, with imbalances linked to periodontitis and other diseases^12^. Thus, understanding the intricate mechanisms and markers of oxidative stress signaling pathways is essential for developing therapeutic strategies aimed at combating oxidative damage and its associated health implications.

p-Synephrine, a natural alkaloid found in bitter orange (Citrus aurantium), has shown potential anti-inflammatory and antioxidant effects, impacting key pathways such as PI3K/AKT, mTOR, NF-κB, and Nrf2. Studies indicate that p-synephrine inhibits the PI3K/AKT signaling pathway, which can reduce the activation of NF-κB^13^. By downregulating NF-κB, p-synephrine can help lower levels of inflammatory markers like IL-6, IL-1β, and TNF-α^14^. Additionally, p-synephrine’s impact on mTOR signaling also contributes to its anti-inflammatory effects^15^, as inhibiting mTOR can further suppress inflammatory responses in immune cells^16^. Moreover, p-synephrine activates the Nrf2 pathway, a key regulator of cellular defense against oxidative damage. Nrf2 activation leads to the upregulation of antioxidant enzymes such as superoxide dismutase (SOD) and catalase, which neutralize reactive oxygen species (ROS) and protect cells from oxidative stress^17^. Overall, p-synephrine’s dual role in modulating both inflammatory and oxidative stress pathways highlights its potential in managing inflammation and oxidative damage-related conditions.

Metal-organic frameworks (MOFs) are versatile porous materials crafted from inorganic ions and organic linkers, designed for high surface area, porosity, and stability. By tailoring structural features like metal clusters and pore sizes, MOFs achieve enhanced active sites and robust stability^18^. NH2-MIL-125, a metal-organic framework (MOF) made of titanium and amino-terephthalic acid, has become a promising material for various biomedical applications due to its large surface area, excellent biocompatibility, and adjustable properties. Extensive research has been conducted on NH2-MIL-125 for its potential in drug delivery, particularly for the controlled and sustained release of therapeutic agents^19^. It can be used to deliver anti-inflammatory and antimicrobial agents directly to the periodontal pocket, enhancing treatment efficacy and reducing side effects associated with systemic drug administration. The controlled release of drugs from NH2-MIL-125 helps maintain therapeutic concentrations at the infection site, improving treatment outcomes and promoting periodontal tissue regeneration^20^.

Exosomes are naturally equipped to deliver a variety of therapeutic agents, including proteins, nucleic acids, and small molecules, due to their excellent biocompatibility and ability to circulate in the body for extended periods^21^. They can be engineered to enhance targeting and delivery efficiency, making them promising candidates for cancer therapy and other diseases^22^.

Exosomes derived from dental pulp stem cells (DPSCs) have emerged as a promising therapeutic tool in the management of periodontal inflammation. These exosomes, which are small extracellular vesicles, play a crucial role in modulating the immune response and promoting tissue regeneration in periodontal diseases. Periodontitis, a common inflammatory disease, leads to the destruction of periodontal tissues and alveolar bone loss due to an immune-inflammatory response to bacterial infection^23^. DPSC-derived exosomes (DPSC-Exo) have been shown to possess anti-inflammatory properties, which are essential in mitigating the effects of periodontitis^24^. Moreover, DPSCs-Exo have demonstrated the ability to enhance the regenerative potential of periodontal tissues. They promote the proliferation, migration, and osteogenic differentiation of periodontal ligament stem cells (PDLSCs), which are crucial for the repair and regeneration of periodontal tissues. This regenerative capability is further supported by their role in suppressing osteoclastogenesis, thereby preventing bone loss associated with periodontitis^25^.

Dexamethasone, a steroid used since the 1960s for inflammatory disorders and certain cancers, is listed on the WHO Model List of Essential Medicines and is widely available off-patent worldwide^26^. This is our reference drug that is going to be used in tandem with p-Synephrine. Lipopolysaccharides (LPS) act as a prototypical endotoxin by interacting with the Cluster of Differentiation 14 (CD14)/Toll-like Receptor 4 (TLR4)/Myeloid Differentiation Factor 2 (MD2) receptor complex, primarily on monocytes, dendritic cells, macrophages, and B cells. This binding triggers the secretion of pro-inflammatory cytokines and nitric oxide^27^. Additionally, LPS promotes oxidative stress and inflammatory responses through the downregulation of Nrf2^28^.

In this study, we aim to assess the therapeutic potential of P-Synephrine loaded onto MOFs and exosomes derived from dental pulp stem cells (DPSCs) in Primary Normal Human gingival keratinocytes (PCS) and Primary human normal gingival fibroblasts (HGF) cells through induction of inflammation and oxidative stress by LPS to examine its efficacy, to reduce inflammation, ultimately lowering the risk of tissue destruction and bone loss over time. The innovation of this study lies in the first bio-inspired comparative evaluation of two distinct advanced delivery systems titanium-based NH_2_-MIL-125 MOF and DPSCs-derived exosomes for targeted carriage of p-synephrine, demonstrating superior efficacy of the exosomal system in simultaneously suppressing the PI3K/mTOR pathway, reducing pro-inflammatory cytokines (IL-4, IL-6, TNF-α), and restoring antioxidant defenses (GPx, SOD, TAC) in an LPS-induced periodontitis model using primary gingival cells.

## Materials and methods

### Reagents

The supplier of the cell lines was Nawah Scientific, located in El Mokattam, Cairo, Egypt. The colorimetric kits, namely the glutathione peroxidase (GPx) kit (Cat. No. GR 25 24), superoxide dismutase (SOD) kit (Cat. No. SD 25 21), and total antioxidant capacity (TAC) kit (Cat. No. TA 25 13), were acquired from Biodiagnostic©, Egypt. In particular, the Interleukin-4 (IL-4) kit (Cat. No. E-EL-H0101), Interleukin-6 (IL-6) kit (Cat. No. E-EL-H6156), Tumor Necrosis Factor Alpha (TNF-α) kit (Cat. No. E-EL-H0109), and Pathways marker ELISA kits were acquired from (SUNLONG BIOTECH CO., LTD ©, China). These immunological assay kits, which are ELISA kits, Mammalian Target of Rapamycin (mTOR) kit (Cat. No. 201-11-4241) and Phosphatidylinositol 3 Kinase (PI3K) kit (Cat. No. SL0571). P-Synephrine (CAS Number: 614-35-7) and LPS (CAS Number: 93572-42-0) were purchased from Sigma Aldrich©.

### Preparation for NH_2_-MIL-125

In order to create NH_2_-MIL-125, titanium isopropoxide (1 mL, 3.38 mmol) and 2-aminoterephthalic acid (1 g, 5.5 mmol) were dissolved at room temperature in a solution of dimethylformamide (DMF)/methanol (2:1 v/v). After being sealed, the resulting slurry was baked for 20 h at 150 °C. The end result was a pale yellow product. After filtering out the product, the unreacted organic ligand was removed by washing it with DMF. The DMF was then exchanged by washing it again with methanol.

### Preparation for p-synephrine

We bought p-synephrine from MAKIN Co. (USA). Adjusted p-synephrine was dissolved in ethanol and water. A 0.22 μm membrane filter (Sigma Aldrich©) was then used to sterilize the p-synephrine solution^29^.

### p-Synephrine loading onto NH_2_-MIL-125

100 mL of ethanol was used to dissolve p-synephrine at varying concentrations (100–1000 ppm) in order to load the medication into NH2-MIL-125 nanoparticles. After adding 1 g of NH2-MIL-125 nanoparticles to the drug solutions, they were agitated for 90 min at room temperature at 600 rpm using a magnetic stirrer. After that, the solution was left alone for the entire night. After five minutes of centrifuging the suspension at 5,000 rpm, the precipitate and supernatant were separated. The difference between the concentration of p-Synephrine in the solution before and after drug loading was used to calculate the amount of loaded drug. The following formula was used to determine the percentage of drug loading:

[(A − B)/A] × 100 is the percentage of drug loading, where A and B stand for the drug solution’s initial and final drug concentrations.

### Isolation of dental pulp stem cells

We gathered caries-free impacted molars from healthy volunteers in the 20–30 age range. Kafrelsheikh University in Egypt’s Committee of Ethics gave its approval for the procedures used to collect the extracted teeth (Ethical approval from Kafrelsheikh University with approval no KFS-272/2025 date 27/5/2025). All methods were performed in accordance with the relevant guidelines and regulations, including the Declaration of Helsinki. Written informed consent for participation and publication of experimental results was obtained from all donors and/or their legal guardians. All procedures involving human tissue were conducted in compliance with institutional ethical standards and donor consent protocols. Following extraction, each tooth was split into multiple pieces using sterile pliers, also known as bone forceps. The teeth’s dental pulp tissue was separated and gathered in an Eppendorf tube. The pulp tissue was cut into 1 × 1 mm2 pieces and digested for 30 to 60 min at 37 °C using a mixture of type IV collagenase (3 mg/ml), as detailed in the earlier study^30^. Next, we obtained a single-cell suspension using a 70 mm cell strainer to filter solutions^30^. After that, the suspension was put onto a 6 cm culture dish and kept in an incubator with 5% carbon dioxide at 37 °C. The culture medium used was the minimum essential medium, which contained 10% fetal bovine serum (FBS, Serena), 100 mg/ml streptomycin (Serena), and 100 units/ml penicillin (Serena). In later studies, we employed the third and fourth passage cells.

### Isolation of exosomes from DPSCs

For the extraction of exosomes, the medium of DPSCs was collected after 48 h of culture, and the centrifugation steps were performed as described previously^31^. In summary, cells were removed from the harvested medium by centrifuging it at 300×g for 10 min and 2000 × g for 10 min. To get rid of cellular debris, the resulting supernatant was then centrifuged for 30 min at 10,000 × g. The resulting supernatant was then centrifuged for 70 min at 100,000×g. The pellet was rinsed with PBS and centrifuged again for 70 min at 100,000 × g (Ultracentrifuge, Beckman Coulter). After discarding the supernatant, the exosome pellets were either cryopreserved at − 80 °C or resuspended in PBS for additional research.

### p-synephrine loaded into exosomes

Sterile water for injection (SWFI) was used to properly dilute P-synephrine (P-SYN) hydrochloride. Triethylamine was used to desalinate the 70 µL of P-SYN HCl (1 mg mL − 1) for one hour at room temperature (RT) after it had been combined with 930 µL of exosome solution (1 mg mL − 1) for 30 min. The Exo-P-SYN complex was then prepared by dialyzing the mix solution against PBS for an entire night at 4 °C in a dialysis tube that had been prepared using a dialysis belt and centrifuge tube^32^.

### Characterization and instrumentation

X-ray diffraction (XRD) patterns were used to characterize the phase purity and crystallinity of the prepared materials (using a Cu Kα monochromator and an X’Pert MPD Philips diffractometer). The scanning electron microscope (SEM: Hitachi SU-70, JP) was used to examine the nanostructure morphology of MOFs. An inverted microscope was used to perform the morphological characterization of dental pulp stem cells (DPSCs). DPSCs had a characteristic spindle-shaped morphology with a noticeable fibroblastic appearance and long cytoplasmic processes at about 70% confluence. The cells created a homogeneous monolayer sheet when they reached full confluence. Using flow cytometry, cells at passage 2 were examined for immunophenotypic analysis. After being separated, DPSCs were rinsed with PBS and incubated with fluorophore-conjugated antibodies that were specific to both non-mesenchymal (CD11-FITC, CD14-PE) and mesenchymal (CD105-PE, CD73-FITC) markers. Flow cytometric analysis was used to validate the cells’ immunophenotypic profile^33^.

Exosome characterization by TEM as previously described^34^ involved isolating the exosome fraction using high-speed centrifugation and fixing it in 2.5% glutaraldehyde/0.1 M sodium cacodylate buffer at 4 °C for 12 h. The pH was stabilized during fixation with 1% OsO4/0.1 M sodium cacodylate buffer. The sample was dehydrated using increasing ethanol concentrations and embedded in epoxy resin. Ultrathin sections were prepared using an ultramicrotome, followed by staining with 1% uranyl acetate and 1% citrate solution. Visualization was performed using a TEM microscope at the central labs of the National Research Centre^35^. Flow cytometry analysis of exosomes was conducted to evaluate surface markers. Exosomes were incubated with mouse anti-human CD9, CD63 and CD81 antibodies for 30 min in 500 µL of flow cytometry staining buffer. The stained exosomes were analyzed using the Attune NxT flow cytometer (Thermo Fisher Scientific). This method confirmed the presence of these characteristic exosome markers, which are critical for validating exosome identity and purity.

### Drug release

P-synephrine from NH_2_-MIL-125 was released in a medium of phosphate buffer saline (pH 7.4) and acetate buffer (pH 4.5). A typical release system was prepared by suspending 2 mg of P-SYN-NH_2_-MIL-125 material in 50 ml of buffer solution (pH 7.4 and 4.5, respectively) at 37 °C. These suspensions were stirred at 200 rpm on a magnetic stirrer for 3 days at 37 °C. A total of 2 ml of release medium was withdrawn at each time point, and UV/Vis spectrophotometry at 339 nm was used to determine the amount of p-synephrine released from NH_2_-MIL-125; after which the sample was returned to the original release system. Each experiment was repeated at least three times. The releasing percentage of P-SYN from P-SYN- NH_2_-MIL-125 was calculated according to the formula, release percentage (%) = *mr/ml*, where *mr* is the amount of released P-SYN, while *ml* is the total amount of loaded P-SYN^36^. NH_2_-MIL-125 is a rigid crystalline metal-organic framework, so its drug release was evaluated using the standard suspension/stirring method in buffer. In contrast, exosomes are soft lipid vesicles that require dialysis to prevent aggregation and maintain structural integrity; therefore, the dialysis bag method was used for Exo-P-SYN.

To measure the in vitro drug release profile, 3 mL of Exo-P-SYN solution was transferred into dialysis tubes immersed in PBS at pH 7.4 or pH 4.5. The release of P-SYN was performed at 37 °C and different time intervals. At 0, 20, 40, 60, 80, 100 and 120 h, the concentrations of P-SYN were determined based on the calibration curve by the *Bioequivalence* ELx800 automated enzyme immunoassay analyzer^32^.

### Cell culture

Primary Normal Human Gingival Keratinocytes (PCS) and Primary human normal gingival fibroblasts (HGF) were obtained commercially from Nawah Scientific (El Mokattam, Cairo, Egypt), a certified provider adhering to ethical sourcing standards. No additional ethics approval was required for their use, as they are established commercial lines. The PCS and HGF cells were cultured in a tissue culture medium, which comprised of DMEM (Serena) 94% supplemented with 5% Fetal Bovine serum (Serena), and 1% penicillin-streptomycin (Serena). Cells were cultured with regular changes of complete media every two days and incubated in a CO_2_ incubator to allow propagation. Once the cells reached 97% confluency, they were sub-cultured using 0.05% trypsin/EDTA (Serena) and continued until reaching the third passage^37^.

### Study design

Cells were divided into eight groups, with each group containing 5–6 × 10⁶ cells cultured in 75 cm² tissue culture flasks. The first group served as the negative control without induction or treatment, while the second group, induced by LPS, acted as the positive control. The third group was induced by LPS and treated with NH_2_-MIL-125, the fourth group was treated with p-synephrine, the fifth group with p-synephrine loaded onto NH_2_-MIL-125, the sixth group with exosomes, the seventh group with p-synephrine loaded into exosomes, and the eighth group with dexamethasone as a reference drug.

### Cell viability

To induce an inflammatory response in Primary Normal Human Gingival Keratinocytes (PCS) and Primary Human Normal Gingival Fibroblasts (HGF), lipopolysaccharides (LPS) (Sigma-Aldrich) were utilized. Following the overnight culture of the cells in a 96-well plate at 37 °C, PCS were exposed to varying concentrations of LPS (0, 0.5, 1, 2, 4, and 8 µg/ml) for 48 h and different concentrations 0, 10, 25, 50, 75, 100 µg/ml of NH_2_-MIL-125, P-SYN, P-SYN-NH_2_-MIL-125, EXO, and P-SYN-EXO, for 72 h. Cell viability was assessed using the MTT assay, where 100 µl of MTT solution (1 mg/ml) was added to each well, followed by a 4-hour incubation at 37 °C. After this period, the supernatants were removed, and 150 µl of DMSO was added to each well to dissolve the formazan crystals, with absorbance measured at 570 nm using an ELX800 microplate reader. For HGF, cells were plated in a 96-well plate and treated similarly with LPS. Absorbance was measured at 570 and 600 nm at specified time points to evaluate the impact of LPS on cell viability. Untreated cells served as a control for both cell types^38^. From analyzing the effects of LPS on cell viability, 2 µg/ml LPS and NH_2_-MIL-125 and 50 µg/ml of P-SYN, P-SYN- NH_2_-MIL-125, EXO, P-SYN-EXO were selected as the optimum doses to use in further experiments and so LPS was added to the cells to induce inflammation in the third subculture, then cells were taken from the culture and were assessed by our kits to see the efficacy of the induction process.

### Treatment

After reaching 5–6 million cells cultured in a 75 cm^2^ flask, and after the third passage, the first group of cells, which are the control group, weren’t treated with any drug and weren’t induced to be used as a negative control. The second group of cells, the positive control group, was induced by LPS only with 2 µg/ml for 48 h. The third group was treated with NH_2_-MIL-125 at 2 µg/mL for 72 h^39^. The fourth group involved the treatment with p-synephrine at 50 µg/mL for 72 h in a dose-dependent manner^40^ The fifth group was treated with P-SYN-NH_2_-MIL-125 at 50 µg/mL for 72 h^41^ The sixth group was treated with exosomes at 50 µg/mL for 72 h, the seventh group was treated with exosomes loaded with p-synephrine at 50 µg/mL for 72 h, and the eighth group was treated for 24 h with dexamethasone at 50 µg/mL for 72 h^42^.

### Biomarker analysis

Immunological analysis was performed using ELISA kits to quantify pro-inflammatory cytokines (IL-4, IL-6, and TNF-α) and pathway markers (PI3K and mTOR). Antioxidant enzyme activities, including Glutathione Peroxidase (GPx), Superoxide Dismutase (SOD), and Total Antioxidant Capacity (TAC), were assessed using colorimetric kits. All procedures were carried out according to the manufacturer’s instructions.

### Statistical analysis

All assays were performed at least three times independently, and the data are presented as the mean ± standard deviation. Differences between groups were assessed by the paired *t*-test (for two-group comparisons) and one-way analysis of variance (ANOVA) followed by Tukey’s post-test (for multiple groups). a: significant difference between control and LPS (*p* < 0.05), b: considerable difference between p-synephrine loaded into NH_2−_MIL-125 and LPS (*p* < 0.01), c: considerable difference between p-synephrine loaded into exosomes and LPS (*p* < 0.01), and d: considerable differences between P-SYN-NH_2−_MIL-125 and P-SYN-Exo (*p* < 0.05).

## Results

### Characterization of NH_2_-MIL-125

**Fig. 1:**
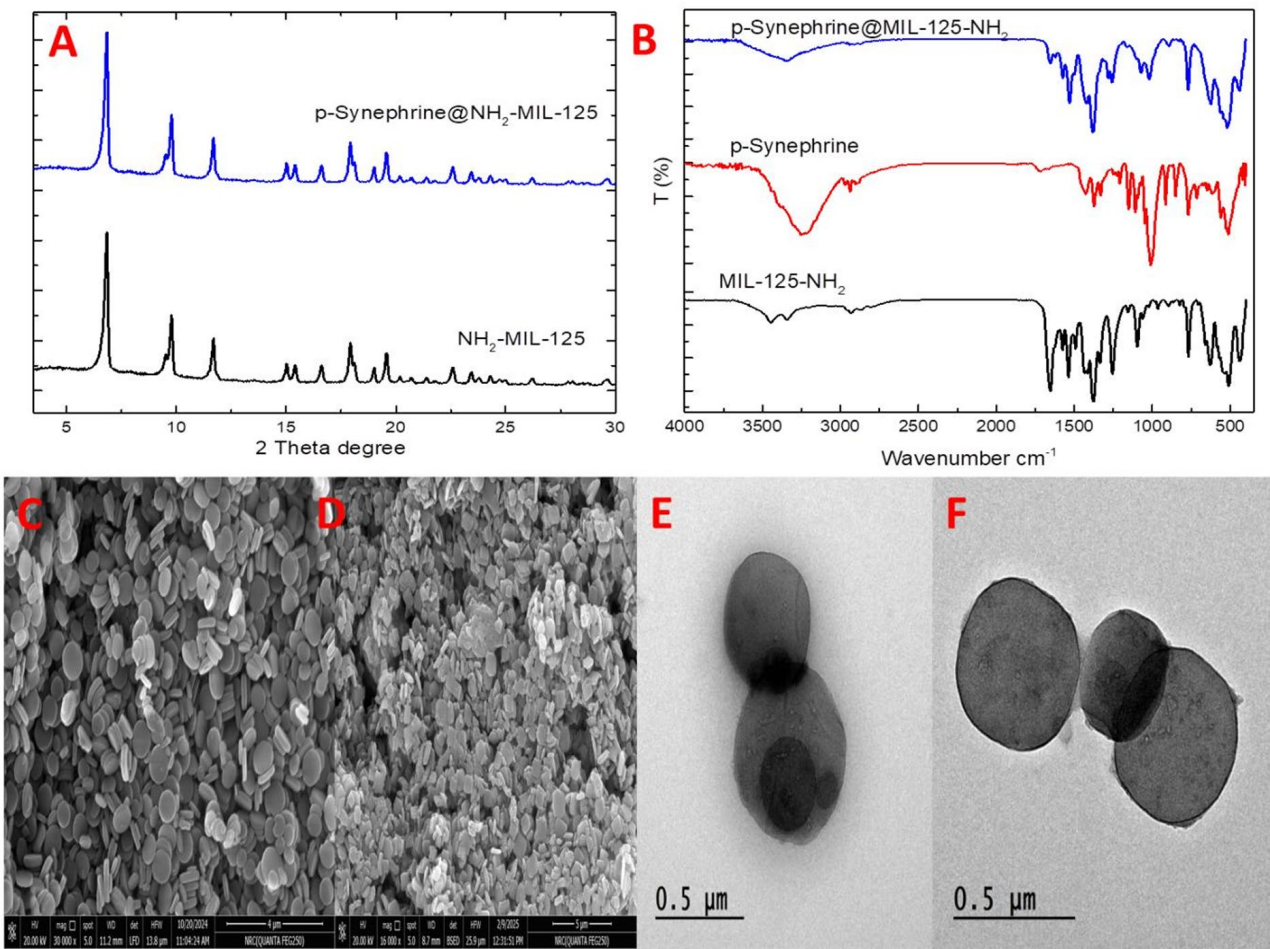
Showing characterization for in which (**A**) is X-ray diffraction patterns of NH_2−_MIL-125 before and after loading, (**B**) FTIR measurements before drug loading and after drug loading, (**C**) is SEM analysis for NH_2−_MIL-125 before drug loading, (**D**) SEM analysis for NH_2−_MIL-125 after drug loading, (E) TEM analysis for NH2-MIL-125 before drug loading and (F) TEM analysis for NH2-MIL-125 after drug loading.

NH_2−_MIL-125 is a complete yellow powder. NH_2_-MIL-125 XRD analysis shows peaks at 6.7, 9.7, 11.6, 15.2, 16.6, 17.9, 19.5, 21.5, 22.6, and 25.3° as shown in Fig. 1(A), which are in good agreement with the simulated PXRD patterns of MIL-125. SEM images of NH_2_-MIL-125 before and after loading are represented in Fig. 1(C and D). They can be seen from the SEM image of NH_2_-MIL-125 before and after loading the dispersed drug on the outer surface of NH_2_-MIL-125. Interestingly, there are no nanoparticles visible in NH_2_-MIL-125 after loading differs from the starting material NH_2_-MIL-125, indicating that the introduction of the drug hasn’t affected NH_2_-MIL-125 morphology, but the interaction between the drug and NH2-MIL-125 can be formed by a chemical bond. FTIR of NH_2_-MIL-125, p- synephrine and drug loading NH_2_-MIL-125 are showed in Fig. 1 (B). NH_2−_MIL-125 FTIR showed bands at at 1576 cm^−1^ corresponds to the vibrational stretching absorption peak of the –C = O group, the peak at 1046 cm^−1^ corresponds to the -OH functional group, and the shoulder peak distributed at 895–676 cm^−1^ is attributed to the C-H bending vibration The O-H stretching vibration peak at 3437 cm^−1^ and the symmetric vibration of the free amino group (-NH_2_) at 3461 cm^−1^. Synephrine FTIR spectrum showed characteristic absorption bands at 1600 –1450 cm⁻¹ region, related to the C = C stretching vibrations. The presence of a hydroxyl group (OH) on the benzene ring will show a broad band in the 3600 –3200 cm⁻¹ region, representing the O-H stretching The amine group (NH) will also exhibit characteristic absorption bands, including a sharp peak in the 3400 –3200 cm⁻¹ region, related to the N-H stretching vibrations, and a band in the 1650 –1500 cm⁻¹ region, representing the N-H bending vibrations. According to the data of Synephrine-NH_2−_MIL-125, the characteristic peak of both materials was observed, confirming successful loading of the drug onto MOFs. Figure 1 (E and F) showing TEM analysis for NH2-MIL-125 before drug loading and after drug loading.

### Characterization of dental pulp stem cells

**Fig. 2:**
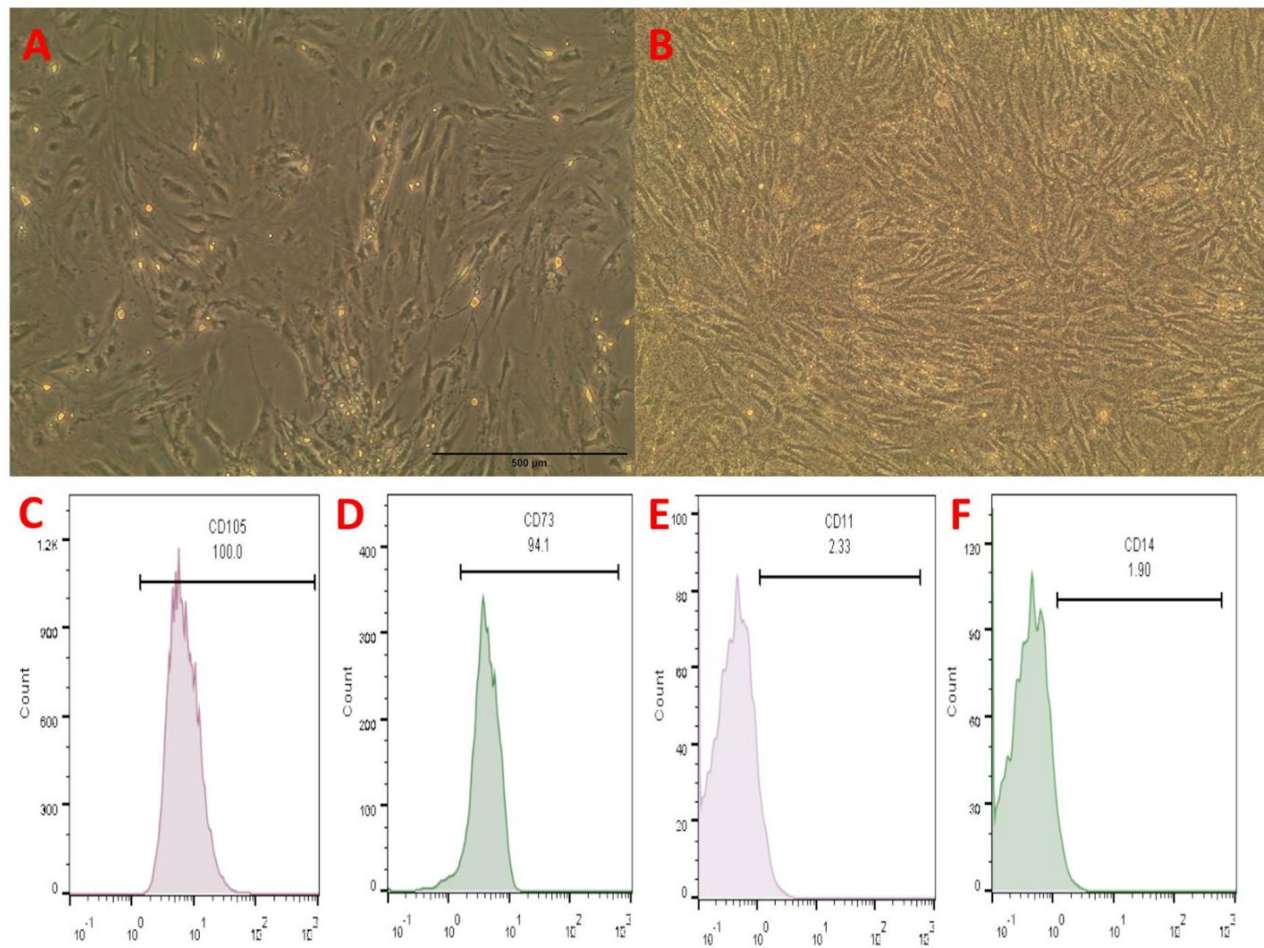
Characterization of dental pulp Stem Cells (DPSCs). (**A**) Cells at 70% confluence showing typical spindle shape. (**B**) Cells at complete confluence forming a monolayer. (**C**) Flow cytometry analysis showed high expression of CD105 (100%) and (**D**) for CD73 (94.1%), (**E**) negative for CD11 (2.33%) and (**F**) for CD14 (1.90%) .

The morphological appearance of DPSCs was observed under an inverted microscope, cells typically exhibit a spindle-shaped, large, and flattened morphology with a pronounced fibroblastic appearance displaying prominent cytoplasmic extensions. Their uniform size and elongated shape indicate healthy proliferative potential as shown in Fig. 2 (A) with a confluence of 70% and in Fig. 2 (B) showing a complete sheet. The flow cytometry showed mesenchymal markers (CD105, CD73) positive in Fig. 2 (C and D) and (CD11, CD14) negative on DPSCs in Fig. 2 (E and F).

### Characterization of DPSCs’ exosomes

**Fig. 3:**
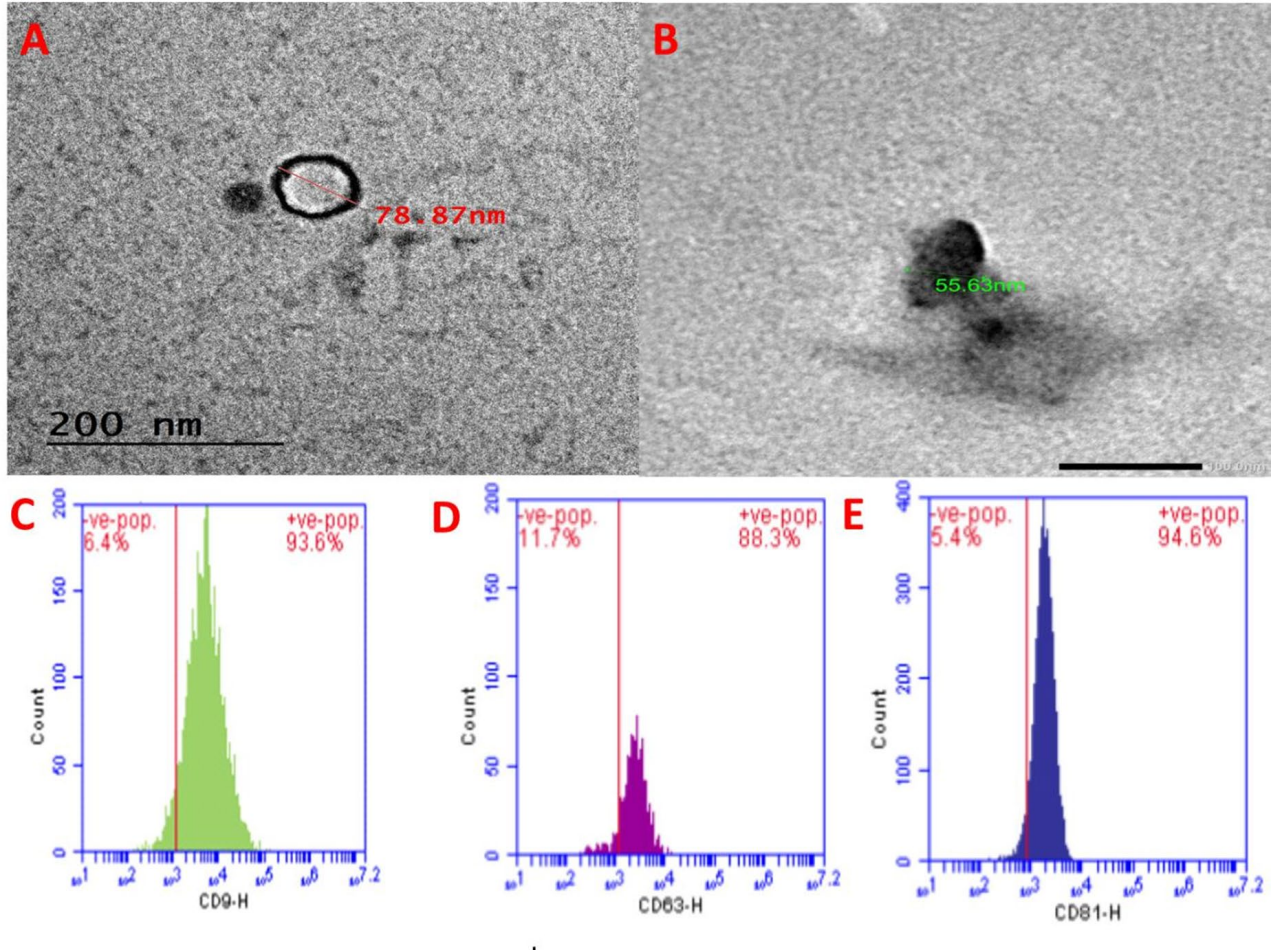
Characterization of DPSCs’ exosomes. (**A**) TEM of exosomes derived from dental pulp stem cells. (**B**) TEM of exosomes loaded with p-synephrine. (**C**) show the CD9 with a positive value of 93.6%, (**D**) show the CD63 with a positive value of 88.3% and (**E**) show the CD81 with a positive value of 94.6%.

Exosomes from dental pulp stem cells (DPSCs) samples were measured via TEM with diameter size 78.87 nm, aligning with the expected size range of 30–150 nm as shown in Fig. 3A. The exosomes display a spherical morphology with well-preserved structural integrity, reflecting the quality of isolation and preparation. TEM of exosomes loaded with p-synephrine with size 55.63 nm was shown in Fig. 3B indicate successful loading without significant aggregation or morphological distortion, suggesting the potential utility of these vesicles as drug delivery systems. Flow cytometry analysis for exosomes was performed to detect the CD surface markers Fig. 3C CD9, Fig. 3D CD63 and Fig. 3E CD81 The exosomes showed positivity to all CD markers.

### MTT assay

**Fig. 4:**
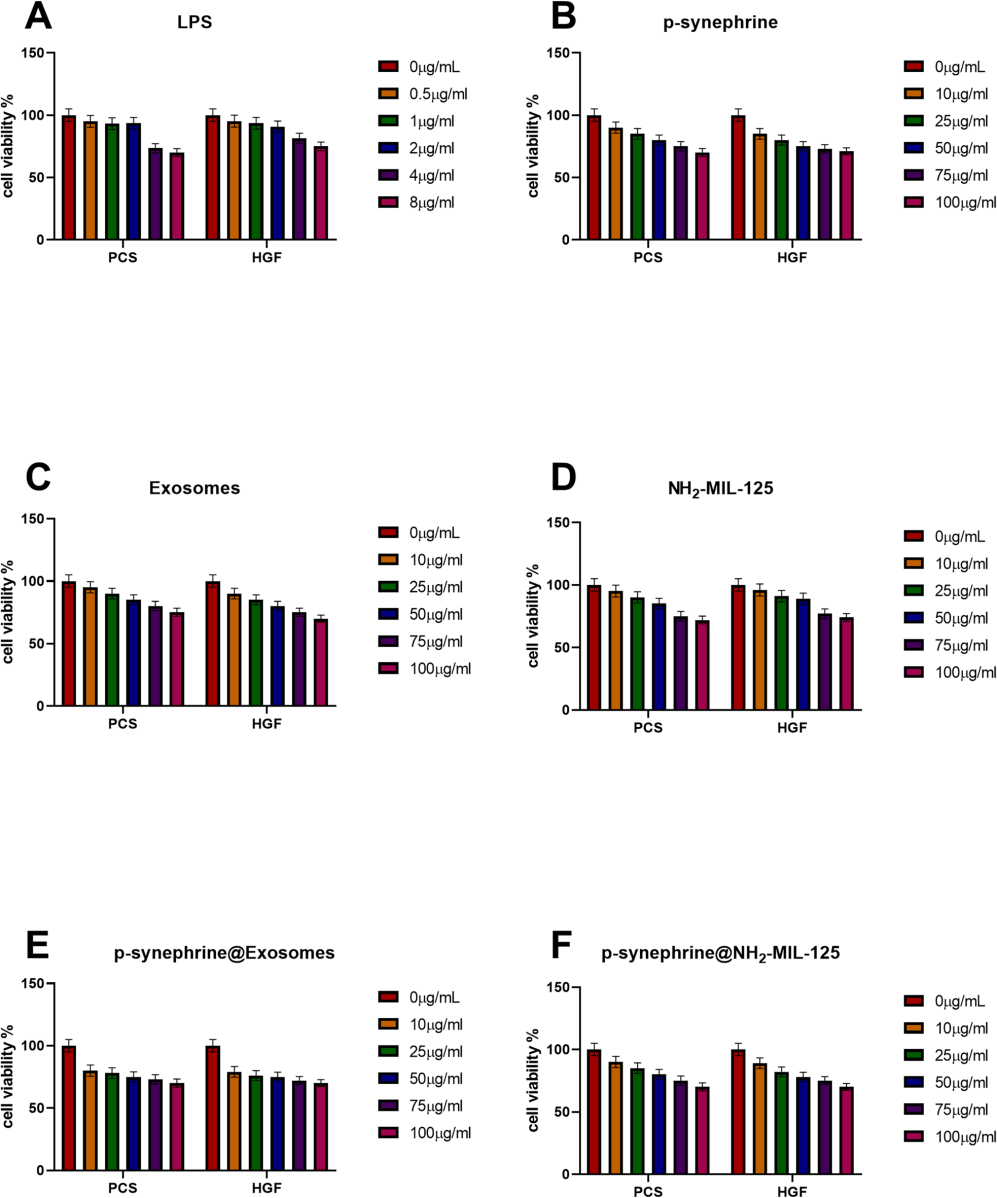
Cell viability tests on PCS and HGF at different concentrations of 0, 10, 25, 50, 75, and 100 µg/ml. (**A**) LPS at different concentrations. (B) p-synephrine at different concentrations. (C) Exosoms at different concentrations. (D) NH_2−_MIL-125 at different concentrations. (E) p-synephrine loaded into NH_2−_MIL-125. (F) p-synephrine loaded into Exosoms. Data are presented as mean ± SD (*n* = 3).

The results demonstrate the impact of LPS, Exosomes, p-synephrine and NH_2−_MIL-125 at varying concentrations on cell viability in PCS and HGF cells in Fig. 4A–D. In PCS cells, a gradual decrease in cell viability is observed with increasing concentrations of both LPS and NH_2−_MIL-125. Similarly, HGF cells exhibit a comparable pattern, with higher concentrations leading to a noticeable reduction in cell viability. Figure 4E illustrates the effects of exosomes loaded with p-synephrine on PCS and HGF cells. For PCS cells, the viability is notably reduced when p-synephrine is loaded into exosomes, compared to using exosomes alone, demonstrating the enhanced effect of the loaded formulation. A similar observation is made in HGF cells, where the loaded exosomes cause a more significant decrease in cell viability. Figure 4F highlights the effects of p-synephrine loaded into NH_2−_MIL-125 on the viability of PCS and HGF cells. In PCS cells, the viability decreases more significantly when p-synephrine is loaded into NH_2−_MIL-125 compared to p-synephrine alone, indicating an enhanced cytotoxic effect. A similar trend is observed in HGF cells, where the loaded compound results in a more pronounced reduction in cell viability than the free compound. LPS treatment caused a significant, concentration-dependent reduction in cell viability in both PCS and HGF cells (*p* < 0.05). Treatment with p-synephrine loaded into NH_2_-MIL-125 or exosomes resulted in a significantly greater decrease in cell viability compared to free p-synephrine or unloaded carriers at 50 µg/ml (*p* < 0.05).

### Drug release

**Fig. 5:**
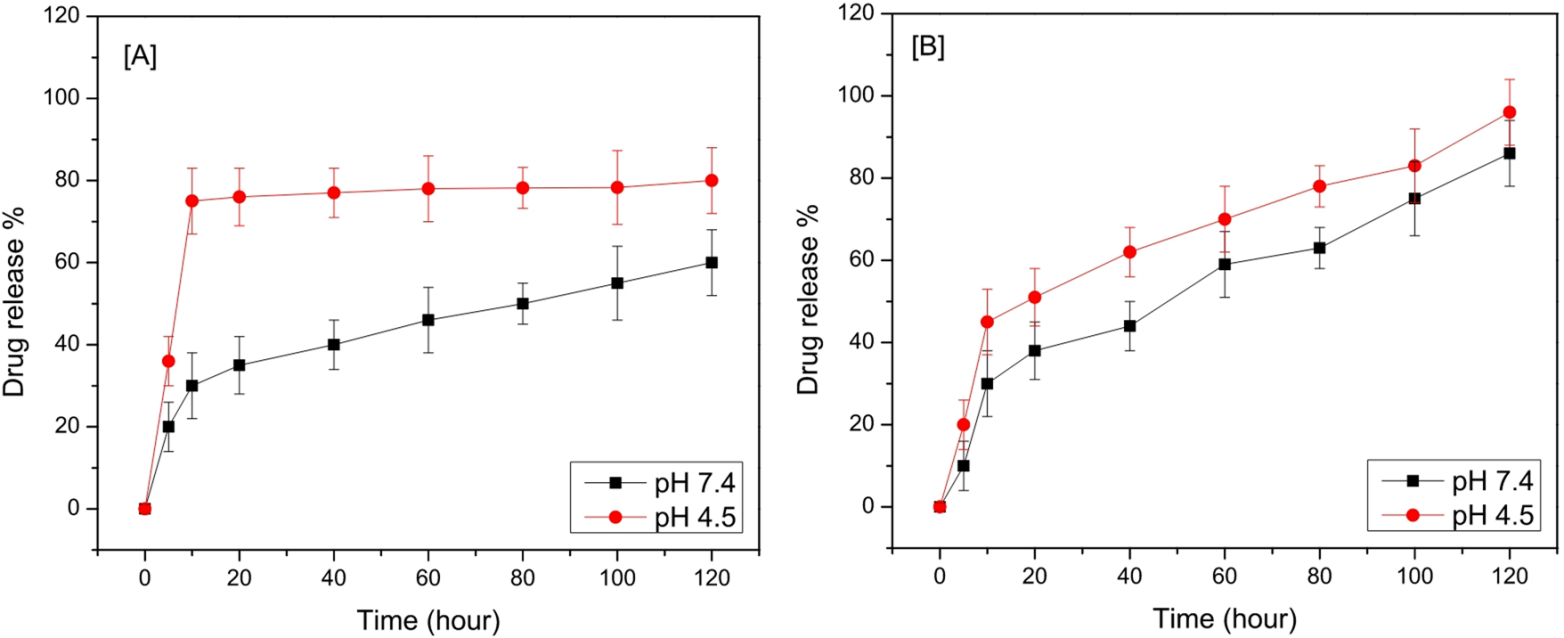
The release profiles of P-SYN. (A) P-SYN release from Exosomes in PBS with pH 4.5 and pH 7.4 and (B) P-SYN release from NH_2−_MIL-125 at pH 4.5 and pH 7.4. Data are presented as mean ± SD (*n* = 3).

To investigate the in vitro p-synephrine drug release profile from Exo-P-SYN and NH_2−_MIL-125-P-SYN, the Exo-P-SYN and NH_2−_MIL-125-P-SYN were placed into PBS dialysate with a pH of 7.4 or 4.5 Fig. 5. In PBS dialysate with pH 7.4, the release of Exo-P-SYN was about 60% after 120 h (Fig. 5A), while in PBS dialysate with pH 4.5, the release rate of Exo-P-SYN boosted rapidly and achieved about 80%, suggesting that the decrease of the pH could result in the protonation of P-SYN and accelerate the release of P-SYN. Figure 5B showed the in vitro release profiles of p-synephrine from NH_2−_MIL-125-P-SYN, at 120 h and pH 4.5, the release of P-SYN is an incomplete and slower value because of NH_2−_MIL-125 has a positive charge surface that reacted with p-synephrine, moreover, a solid-state grafting reaction also effect on the release profile. Additionally, the electrostatic interaction between the grafted p-synephrine and NH_2−_MIL-125 can stop the dissociation and cause slow release. In conclusion, the loading amount of P-SYN is 19 (w/w) % and the obtained data confirmed that NH_2−_MIL-125 can be used as effective drug carriers.

### Biomarkers results

#### Inflammatory and oxidative stress markers

**Fig. 6:**
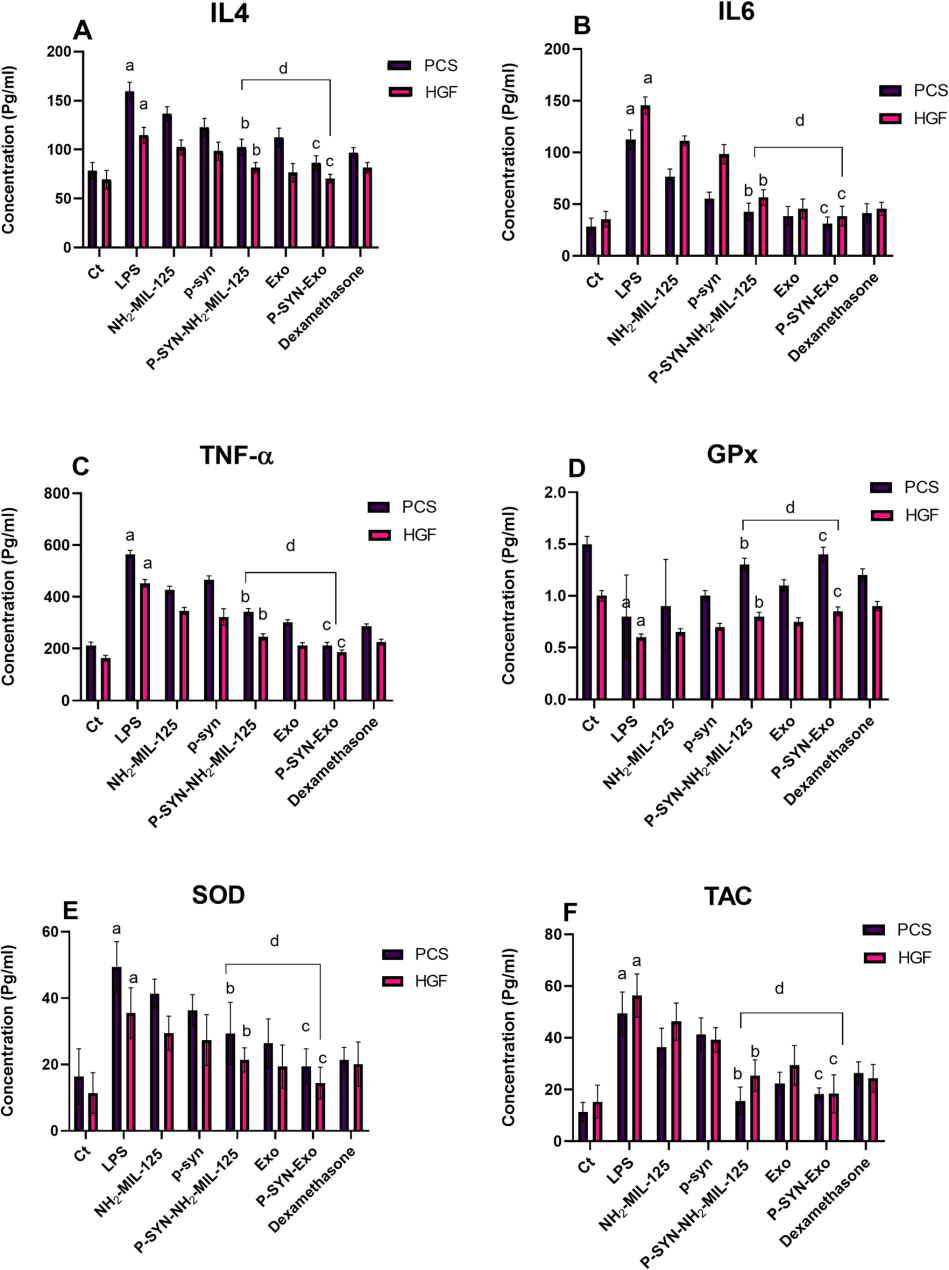
Showing the ELISA and colorimetric analysis. ELISA results of interleukins (IL4 (A), IL6 (B)) and tumor necrosis factor-alpha (TNF-α) (C) of the primary normal human gingival keratinocytes (PCS) and primary normal human gingival fibroblast (HGF) that induced with lipopolysaccharide and treated with different groups of NH_2−_MIL-125, P-synephrine, P-synephrine loaded into NH_2−_MIL-125 (P-SYN- NH_2−_MIL-125), exosomes derived from DPSCs, P-synephrine loaded into exosomes (P-SYN-Exo), and dexamethasone as a reference drug. Colorimetric assay results for antioxidant markers (D) GPx, (E) SOD, and (F) TAC in PCS and HGF cells. Data are presented as mean ± SD (*n* = 3).a: significant difference between control and LPS (*p* < 0.05), b: considerable difference between P-synephrine loaded into NH_2−_MIL-125 and LPS (*p* < 0.01), and c: considerable difference between p-synephrine loaded into exosomes and LPS (*p* < 0.01) and d: considerable differences between P-SYN- NH_2−_MIL-125 and P-SYN-Exo (*p* < 0.05).

The results demonstrate that stimulation with LPS significantly increased the levels of IL-4, IL-6, and TNF-α in both PCS and HGF cells compared to the control group. Treatment with NH_2−_MIL-125 and p-synephrine effectively reduced these levels, with a more pronounced anti-inflammatory effect observed for P-SYN-NH_2−_MIL-125. Furthermore, treatment with exosomes alone and p-synephrine-loaded exosomes (P-SYN-Exo) exhibited significant anti-inflammatory effects, reducing IL-4, IL-6, and TNF-α levels. Notably, P-SYN-Exo demonstrated a stronger effect compared to P-SYN-NH_2−_MIL-125, indicating that the encapsulation into exosomes enhances its bioavailability and anti-inflammatory potential. Among all treatments, P-SYN-NH_2−_MIL-125 and P-SYN-Exo exhibited the strongest anti-inflammatory effect, as evidenced by the lowest levels of all three inflammatory markers, as shown in Fig. 6. These findings highlight the potential of P-SYN-NH_2−_MIL-125 and p-synephrine-loaded exosomes as promising therapeutic agents for managing inflammation, particularly in comparison to traditional anti-inflammatory treatments with dexamethasone. In colorimetric assay the results demonstrate the effects of different treatments on antioxidant markers (GPx, SOD, and TAC) in PCS and HGF cells under oxidative stress induced by LPS. LPS treatment significantly suppressed the levels of GPx, SOD, and TAC, highlighting oxidative damage. Treatments with NH_2−_MIL-125 and free p-synephrine partially restored antioxidant enzyme activities, but p-synephrine loaded into NH_2−_MIL-125 (P-SYN-NH_2−_MIL-125) and exosomes (P-SYN-Exo) showed superior recovery, with P-SYN-Exo demonstrating the most notable enhancement across all markers. This effect was comparable to the standard anti-inflammatory agent dexamethasone, particularly for P-SYN-Exo, which exhibited the highest restoration of GPx, SOD, and TAC levels as shown in Fig. 6. These findings suggest that p-synephrine encapsulated in advanced delivery systems like NH_2−_MIL-125 and exosomes significantly improves the restoration of antioxidant defenses, likely due to enhanced bioavailability and sustained release, presenting a promising therapeutic approach against oxidative stress.

#### Pathway markers

**Fig. 7:**
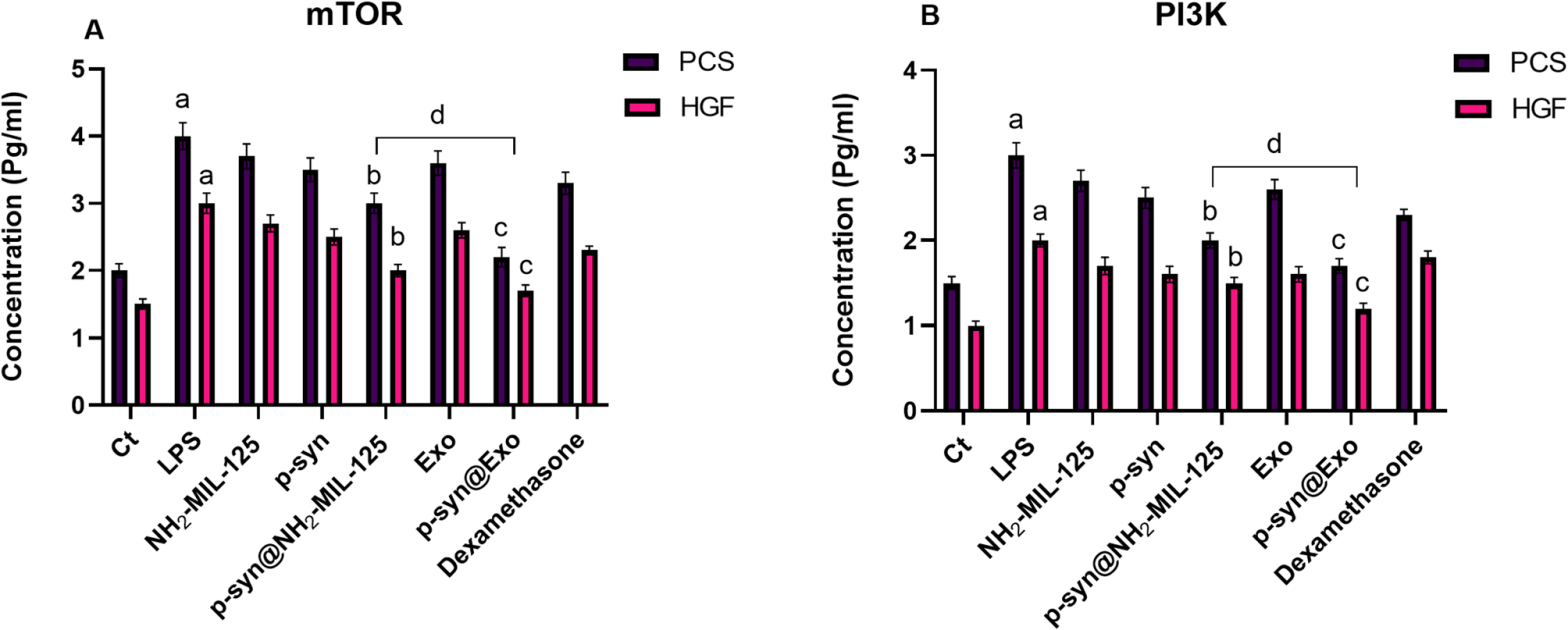
The concentrations of (A) Mammalian target of rapamycin (mTOR) and (B) phosphatidylinositol 3 kinases (PI3K) of the primary normal human gingival keratinocytes (PCS) and primary normal human gingival fibroblast (HGF) that induced with lipopolysaccharide and treated with different groups of NH_2−_MIL-125, P-synephrine, P-synephrine loaded into NH_2−_MIL-125 (P-SYN-NH_2−_MIL-125), exosomes derived from DPSCs, P-synephrine loaded into exosomes (P-SYN-Exo), and dexamethasone as a reference drug, a: significant difference between control and LPS (*p* < 0.05), b: considerable difference between p-synephrine loaded into NH_2−_MIL-125 and LPS (*p* < 0.01), c: considerable difference between p-synephrine loaded into exosomes and LPS (*p* < 0.01), and d: considerable differences between P-SYN- NH_2−_MIL-125 and P-SYN-Exo (*p* < 0.05). Data are presented as mean ± SD (*n* = 3).

The results highlight the effects of various treatments on the pathway marker mTOR in Primary Gingival Keratinocytes (PCS) and Gingival Fibroblasts (HGF) cells. Lipopolysaccharide (LPS) stimulation significantly increased PI3K and mTOR levels, indicating the activation of inflammatory pathways. Treatments with NH_2−_MIL-125 and free p-synephrine moderately reduced these markers, while p-synephrine loaded into NH_2−_MIL-125 (P-SYN- NH_2−_MIL-125) demonstrated an inhibitory effect, reflecting the enhanced efficacy of the encapsulated formulation. Exosomes and p-synephrine-loaded exosomes (P-SYN-Exo) further a stronger reduced PI3K and mTOR levels, with P-SYN-Exo showing superior suppression, likely due to improved bioavailability and targeted delivery. P-SYN-NH_2−_MIL-125 and P-SYN-Exo exhibited the most substantial reduction in PI3K and mTOR, as shown in Fig. 7. These findings underscore the potential of nanocarrier systems such as NH_2−_MIL-125 and exosomes to enhance the therapeutic effects of anti-inflammatory agents.

## Discussion

As we know, periodontitis is a common inflammatory disease driven by bacterial infection and an overactive immune response, leading to the destruction of tooth-supporting tissues and potential tooth loss. Excessive reactive oxygen species (ROS) from hyperactive neutrophils, which occur as a side effect to inflammation, damage tissues and can overwhelm antioxidant defenses^43^. Additionally, bacteria and inflammatory factors from the gums can enter the bloodstream, contributing to systemic inflammation and potentially impacting conditions like cardiovascular disease, diabetes, and chronic kidney disease^44^. p-Synephrine was loaded onto NH_2_-MIL-125 (a biocompatible titanium-based MOF known for its high porosity, pH-responsive controlled release, and excellent stability) and DPSCs-derived exosomes (natural nanoscale vesicles with superior biocompatibility, cellular uptake, and targeting ability) to compare two fundamentally different advanced delivery platforms. The significance of this comparison lies in evaluating a synthetic inorganic carrier versus a biological nanocarrier for enhancing the bioavailability, sustained release, and therapeutic efficacy of p-synephrine in mitigating LPS-induced inflammation and oxidative stress in primary gingival cells.

Moreover, periodontal disease management primarily involves non-surgical treatments, such as scaling, root planning, and root debridement, which help reduce inflammation and prevent further tissue damage, and we should keep in mind that proper oral hygiene, smoking cessation, and lifestyle changes are essential for effective outcomes, also manual and powered brushes, as well as interdental brushes, aid in plaque control, patient education on usage is key^45^. On the other hand, antibiotics and local antimicrobials are generally reserved for aggressive cases to minimize resistance risks. Newer adjuncts like lasers and host-modulatory drugs show promise but need more evidence before becoming standard practice^46^.

In periodontal disease, Toll-like receptors (TLRs) on immune and gingival epithelial cells detect pathogen-associated molecular patterns (PAMPs), such as lipopolysaccharides (LPS), and trigger an inflammatory response via the NF-κB signaling pathway. This pathway operates through classical and nonclassical routs, with the classical pathway activated when LPS binds to TLR4, leading to IκBα phosphorylation and degradation, releasing the NF-κB p65/p50 complex, which then translocate into the nucleus to suppress osteogenic markers like OPN, OCN, Osx, and Runx2 in periodontal ligament stem cells (PDLSCs), impairing bone regeneration and promoting bone loss^47^. Additionally, NF-κB activation induces inflammatory cytokines such as TNF-α, IL-6, and IL-1β, further amplifying inflammation and contributing to periodontitis pathogenesis^48^. Following cytokine release, PI3K is recruited to the cell membrane, where it helps convert PIP2 to PIP3^49^, and together with mTORC2, activates AKT, which promotes cell survival by inhibiting pro-apoptotic proteins like Bad and enhancing anti-apoptotic factors such as Bcl-2^50^. AKT also activates mTOR, a key regulator in protein synthesis, lipid metabolism, and cell growth, existing in two complexes: mTORC1 and mTORC2. While mTORC1 controls metabolic processes, mTORC2 is essential for AKT activation and cytoskeletal organization^51^, also modulating NF-κB signaling to increase inflammatory cytokine production, thus highlighting the interconnectedness of these pathways in inflammation, homeostasis, and immune surveillance^52^.

Lipopolysaccharides (LPS) are key outer membrane components of gram-negative bacteria. Structurally, they are amphipathic glycoconjugates made up of a lipid domain (hydrophobic) linked to a core oligosaccharide and a distal polysaccharide^53^. LPS activates innate immune cells like macrophages and neutrophils, leading to the production of proinflammatory factors, such as IL-1β, TNF, MMPs, and free radicals, which contribute to significant secondary inflammation in tissues^54^. LPS also induces oxidative stress, notably decreasing TAC and GPx activities in serum and liver while significantly raising serum MDA levels^55^.

P-synephrine’s antioxidant effects may help protect against periodontitis by reducing oxidative stress. By boosting antioxidant enzymes like SOD and catalase, p-synephrine can lower ROS levels, which are major contributors to inflammation and tissue damage in periodontal disease^56^. This reduction in ROS could also stabilize AKT and mTOR pathways, preserving cell survival and balancing immune responses, Additionally, p-synephrine inhibits Eotaxin-1 production by suppressing STAT6 phosphorylation and translocation in IL-4-induced cells, reducing eosinophil recruitment and further mitigating inflammation^57^. Ishida et al. mentioned that p-synephrine significantly reduced the mRNA expression of inducible nitric oxide synthase (iNOS), an enzyme responsible for nitric oxide production. However, Ishida et al. also mentioned that p-synephrine did not alter the mRNA expression of cyclooxygenase-2 (COX-2), a critical enzyme in prostaglandin synthesis^29^.

The recent advances in nanometal-organic frameworks (MOFs) for drug delivery include various new types of frameworks such as MIL-53, MOF-74, and UiO-66, among others. These MOFs, utilizing templates like HKUST, UiO, ZIF, and MIL, offer diverse cargo delivery methods such as encapsulation, direct assembly, and post-synthesis strategies^58^. They are effective carriers for drugs like doxorubicin^59^, 5-fluorouracil^60^, and curcumin^61^, demonstrating distinct stability profiles in biological environments. MOFs show promise over traditional carriers like carbon nanotubes and gold nanoparticles by enabling precise drug loading and controlled release at the atomic level^62^. This shows the possibility of MOFs combining P-synephrine and being used as a drug carrier.

In our experimental work, we illustrate the inflammatory and oxidative mechanisms that involve various markers that undergo significant changes. Pro-inflammatory cytokines such as IL-4, IL-6, and TNF-α are markedly elevated due to the activation of the NF-κB pathway in response to lipopolysaccharides (LPS) in periodontal disease. This increased cytokine production exacerbates inflammation, leading to a cascade of immune responses and tissue damage. NF-κB is activated, promoting the transcription of genes responsible for inflammatory mediators, further amplifying the inflammatory process. The PI3K/AKT pathway, which is activated by inflammatory cytokines, further contributes to the inflammatory state by promoting cell survival and proliferation while enhancing the inflammatory response^63^. Notably, mTOR, influenced by AKT, also plays a role in this network by regulating protein synthesis and immune responses^64^.

The treatments tested in our experiment showed similar results to other articles where p-synephrine demonstrates promising anti-inflammatory and antioxidant properties. By inhibiting the PI3K/mTOR pathway, it downregulates NF-κB activity, leading to reduced levels of pro-inflammatory cytokines, such as IL-4, IL-6 and TNF-α^65^. Additionally, p-Synephrine increased intracellular GSH concentration and GPx enzymatic activity however, it didn’t have an effect on SOD and catalase, but still managed to mitigate oxidative stress by lowering ROS levels^66^. This dual mechanism decreases inflammation and fosters a more stable cellular environment, potentially improving periodontal health.

The incorporation of p-Synephrine into NH_2−_MIL-125 could amplify its antioxidant and anti-inflammatory actions through sustained release and increased bioavailability, further reducing levels of IL-4, IL-6, and TNF-α and increasing antioxidative stress markers of GPx, SOD, and TAC. But the p-synephrine which loaded into exosomes (P-SYN-Exo) showed a stronger reduction in inflammation markers of IL-4, IL-6, TNF-α, and inflammation pathway markers of PI3K and mTOR and increasing in antioxidant markers of GPx, SOD, TAC than p-synephrine loaded in NH_2−_MIL-125 (P-SYN-NH_2−_MIL-125). The increased effectiveness of p-synephrine when encapsulated in exosomes, as seen in our research, is corroborated by studies demonstrating that exosomes are capable of efficiently transporting therapeutic miRNAs to influence inflammatory and oxidative stress pathways^67^. A study featured in the *Journal of Neurological Diseases* indicates that exosomes can boost the levels of antioxidant factors and anti-inflammatory agents, including IL-4 and IL-10, while simultaneously decreasing pro-inflammatory elements such as IL-6 and TNF-α, by influencing the PI3K/Akt/mTOR pathway^68^.

Exosomes loaded with anti-inflammatory drugs, such as mycophenolic acid and rosmarinic acid, have demonstrated superior anti-inflammatory effects compared to free drugs. These exosomes modulate inflammatory pathways, such as TLR4-NLRP3, reducing cytokine production and inflammation in various models^69,70^ and this is compatible with our study of the exosomes loaded with p-synephrine have demonstrated a significant decrease in inflammation markers of IL-4, IL-6, and TNF-α compared to free p-synephrine.

Exosomes loaded with antioxidants, such as selenium, have demonstrated a remarkable ability to reduce oxidative stress markers and elevate antioxidant levels in diabetic models, leading to improved liver function and diminished oxidative damage^71^. These findings align closely with our results, which reveal that exosomes loaded with p-synephrine significantly enhance antioxidative markers, including GPx, SOD, and TAC. Furthermore, exosomes derived from adipose-derived stem cells have been shown to regulate the Nrf2/HO-1 axis, thereby enhancing antioxidative responses and reducing oxidative stress in macrophages, a mechanism particularly beneficial in conditions such as sepsis^72^. This improvement underscores the potential of exosome-based delivery systems in amplifying the bioavailability and efficacy of antioxidants, thereby mitigating oxidative stress and promoting cellular protection. Such outcomes not only validate the efficacy of exosome-mediated antioxidant delivery but also highlight the therapeutic potential of p-synephrine-loaded exosomes in combating oxidative stress-related conditions.

## Conclusion

This study highlights the potential of p-Synephrine, NH_2−_MIL-125, and exosome-based delivery systems as effective therapeutic agents in managing inflammation and oxidative stress in periodontal disease. Periodontitis, driven by bacterial infection and a heightened immune response, exacerbates tissue damage through increased ROS levels and inflammatory cytokines. Our findings suggest that p-Synephrine’s inhibition of the PI3K/ mTOR pathway and its activation of antioxidant defenses, increased SOD, TAC, and GPx activity, contribute to reduced inflammation. Furthermore, NH2-MIL-125’s role as a controlled-release carrier for p-Synephrine offers additional therapeutic benefits, enhancing p-Synephrine’s bioavailability and sustaining its release to maintain therapeutic levels. Similarly, p-Synephrine-loaded exosomes derived from DPSCs further amplified these effects by providing targeted delivery, improved cellular uptake, and enhanced bioavailability. These exosomes demonstrated superior anti-inflammatory and antioxidant properties, significantly reducing cytokine levels of TNF-α, IL-4, and IL-6 while increasing the antioxidative stress of GPx, TAC, and SOD. The dual use of p-Synephrine’s anti-inflammatory and antioxidant mechanisms, alongside NH_2−_MIL-125 and exosome-controlled release capabilities, presents a promising approach for reducing cytokine levels, balancing oxidative stress, and mitigating periodontal tissue degradation.

## Limitations

This study is limited to in vitro experiments using two primary cell lines (PCS and HGF) and LPS-induced inflammation as a simulated periodontitis model. The findings require validation in animal models of periodontitis and eventually in clinical settings. Additionally, the study did not investigate long-term stability of the formulations, potential immune responses to the carriers, or detailed molecular mechanisms beyond PI3K/mTOR and selected oxidative stress markers. No in vivo biodistribution or toxicity studies were performed.

## Data Availability

Data are available on reasonable request from the authors.

## Abbreviations

COX-2: Cyclooxygenase-2
DMF: Dimethylformamide
DPSCs: Dental pulp stem cells
ELISA: Enzyme-Linked Immunosorbent Assay
GPx: Glutathione peroxidase
HGF: Primary human normal gingival fibroblasts
IL-4: Interleukin 4
iNOS: Inducible nitric oxide synthase
LPS: Lipopolysaccharide
MD2: Myeloid differentiation factor 2
MOF: Metal organic frame
mTOR: Mammalian target of rapamycin
PAMPs: Pathogen-associated molecular patterns
PCS: Primary normal human gingival keratinocytes
PDLSCs: Differentiation of periodontal ligament stem cells
PI3K: Phosphatidylinositol 3 kinase
PMPR: Professional mechanical plaque removal
P-SYN: P-Synephrine
ROS: Reactive oxygen species
SOD: Superoxide dismutase
SWFI: Sterile water for injection
TAC: Total antioxidant capacity
TEM: Transmission electron microscope
TLR4: Toll-like receptor 4
TNF-α: Tumor necrosis factor alpha
XRD: X-ray diffraction
